# Salivary Cortisol and Alpha-amylase—Biomarkers of Stress in Children undergoing Extraction: An *in vivo* Study

**DOI:** 10.5005/jp-journals-10005-1514

**Published:** 2018-06-01

**Authors:** Yogita Chaturvedi, Shefali Chaturvedy, Nikhil Marwah, Saurabh Chaturvedi, Swati Agarwal, Neha Agarwal

**Affiliations:** 1 Postgraduate Student, Department of Pedodontics and Preventive Dentistry Mahatma Gandhi Dental College & Hospital, Jaipur, Rajasthan India; 2 Reader, Department of Pedodontics and Preventive Dentistry Mahatma Gandhi Dental College & Hospital, Jaipur, Rajasthan India; 3 Professor and Head, Department of Pedodontics and Preventive Dentistry Mahatma Gandhi Dental College & Hospital, Jaipur, Rajasthan India; 4 Reader, Department of Orthodontics and Dentofacial Orthopedics Government Dental College, Jaipur, Rajasthan, India; 5 Postgraduate Student, Department of Pedodontics and Preventive Dentistry Mahatma Gandhi Dental College & Hospital, Jaipur, Rajasthan India; 6 Postgraduate Student, Department of Pedodontics and Preventive Dentistry Mahatma Gandhi Dental College & Hospital, Jaipur, Rajasthan India

**Keywords:** Saliva, Salivary alpha-amylase, Stress.

## Abstract

**Aim:**

The anxiety of dental procedure evokes physiological response in the human body similar to fear. The level of cortisol and alpha-amylase in saliva can be considered as one of the major biomarkers of stress and anxiety. Our study was aimed to correlate the stress and anxiety with the levels of salivary cortisol (SC) and salivary alpha-amylase (SAA) in patients undergoing routine dental extraction.

**Materials and methods:**

The levels of SC and SAA were assessed pre- and postextraction in the salivary samples of 20 children.

**Results:**

The values of cortisol and alpha-amylase showed a significant increase postextraction.

**Conclusion:**

Salivary cortisol and SAA can be considered an important and noninvasive tool for assessment of anxiety, such as dental extraction, in children.

**Clinical significance:**

Increase in the stress levels of a child in the dental operatory procedures like tooth extraction suggests the use of some behavior modification and shaping techniques by dentists to overcome the anxiety of the child before commencement of the procedure. This can aid in better cooperation of the child during treatment as well as helps in internal motivation toward future dental treatment.

**How to cite this article:** Chaturvedi Y, Chaturvedy S, Marwah N, Chaturvedi S, Agarwal S, Agarwal N. Salivary Cortisol and Alpha-amylase—Biomarkers of Stress in Children undergoing Extraction: An *in vivo* Study. Int J Clin Pediatr Dent 2018; 11(3):214-218.

## INTRODUCTION

The unknown, be it a person, thing, or procedure, evokes fear in the human mind. By observing other people and learning by their or our own experiences, we try to cope with our surroundings.

The anxiety of dental procedure also evokes physiological response in the human body similar to fear. Bandura suggests that the child patient along with parents should closely watch an already adapted child being treated in the dental operatory.^1^ Other methods suggested to combat this stress in patients are hypnosis and use of medications.^2^

Subjective assessment of this stress is of limited value, as it is incorporated with inherent human bias. Hence, evaluation of various factors like adrenocorticotropic hormone (ACTH), serotonin, and cortisol in serum is recommended. But the mere sight of blood and the act of its withdrawal from the body can induce stress and hence, influence the readings.

Analysis of one’s saliva can fortunately overcome these limitations. In the last decade, saliva has gained importance as an important diagnostic aid partially due to its abundance of biomarkers and due to its ease and noninvasive accessibility. Cortisol plays a major role in the stress response.

An increase in the production of serum cortisol by adrenal cortex leads to a proportionate increase in its level in saliva. Hence, SC concentrations are closely correlated to serum cortisol concentration.^3,4^ Under the influence of sympathetic stimuli, salivary glands secrete SAA, which is one of the major salivary enzymes in humans.^5^

A correlation was reported by Chatterton et al^6^ between the concentration of salivary amylase and blood levels of catecholamines. Hence, the measure of SAA can give a clue regarding the level of stress. The aim of our study was to evaluate the values of biomarkers, SAA and SC and their correlation, pre- and postdental extraction, in children that may represent a stressful event in children without previous dental clinic exposure.

## MATERIALS AND METHODS

A cross-sectional study was designed and the sample consisted of randomly selected 20 patients who reported to the Department of Pedodontics and Preventive Dentistry, Mahatma Gandhi Dental College & Hospital, between 5 and 12 years of age and had deciduous teeth indicated for dental extractions. A written consent, after thorough explanation about the procedure, was obtained from the subjects/parents.

**Fig. 1:**
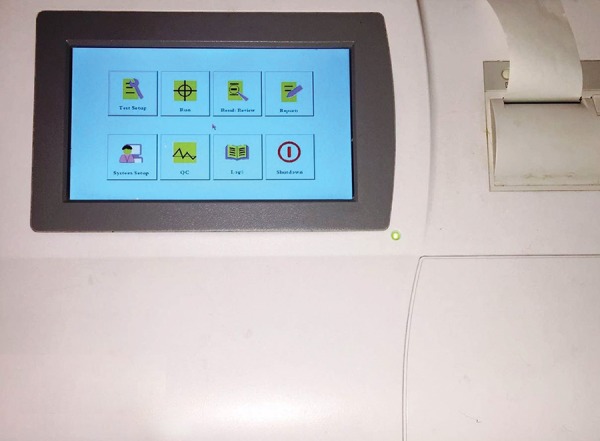
The new life Cortisol ELISA kit

**Fig. 2:**
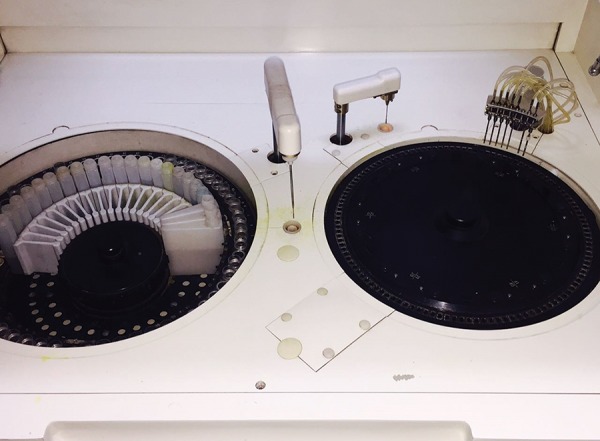
Spinreact S.A/S.A.U CNPG3

Exclusion criteria included physically and medically compromised conditions, subjects with arrested carious lesions, medications, especially corticosteroids. For the collection of saliva, the child was seated in the Coachman’s position, in which head was bent slightly down and the child was asked not to swallow or move his tongue or lips during the period of collection.

The patient was asked to pool the saliva in his/her mouth for 5 minutes and then to drool it passively in the receiving vessel. Two samples of unstimulated saliva were collected with this method from each subject, TO: after initial screening and outpatient department (OPD) of the subject and T1: 30 minutes postextraction and after the cessation of bleeding. To rule out any bias occurring due to diurnal variation, all the samples were collected at the same time, i.e., between 10 am and 1 pm; 2 mL saliva from each sample was taken and stored at -20° till evaluation.

The SC level was measured with the help of “The new life Cortisol ELISA kit” (Fig. 1) which utilizes solid-phase competitive enzyme-linked immunosorbent assay (ELISA).

The level of alpha-amylase in saliva was measured on “Spinreact S.A/S.A.U CNPG3” (Fig. 2). All analyses were performed utilizing Statistical Package for the Social Sciences software. The means and standard deviations (SDs) were calculated each for SAA pre-extraction (SATO), SAA postextraction (SAT1), SC pre-extraction (SCT0), SC postextraction (SCT1), and also for the difference in values at TO and T1.

Pearson’s correlation coefficient was used to correlate between changes in SC and SAA levels.

**Table 1:** **Table 1:** SAA and SC values pre- and postextraction

		*T0*		*T1*		*Difference*			
		*Mean± SD*		*Mean ± SD*		*Mean± SD*		*p-value < 0.05*	
SAA		0.60285		1.069		0.4671		0.00609	
		0.8767		0.9868		0.3310			
SC		15.4325		27.18		11.75		0.000023	
		6.565		9.347		7.1795			

## RESULTS

Table 1 compares between SAA and SC values pre- and postextraction. For SATO, mean 0.602, SD 0.876, and SAT1 mean 1.06995, SD 0.986. The mean increase was 0.45, which was statistically significant (p = 0.006 < 0.05). For SCT0, mean 15.4325, SD 6.565, and SCT1, mean 27.18, SD 9.347. It also depicted the changes in the postextraction values.

**Graph 1:**
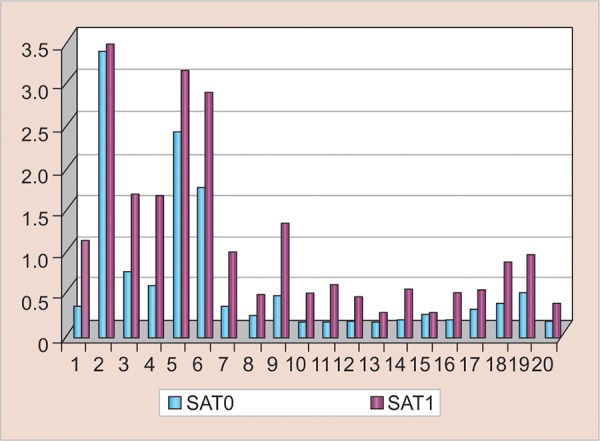
Comparison between SAA pre- and postextraction

The mean increase was 7.179, which was highly statistically significant (p = 0.000023 < 0.05). Graph 1 shows the comparison between SAT0 (mean 0.602, SD 0.876) and SAT1 (mean 1.06995, SD 0.986). The mean increase was 0.45, which was statistically significant (p = 0.006 < 0.05).

**Graph 2:**
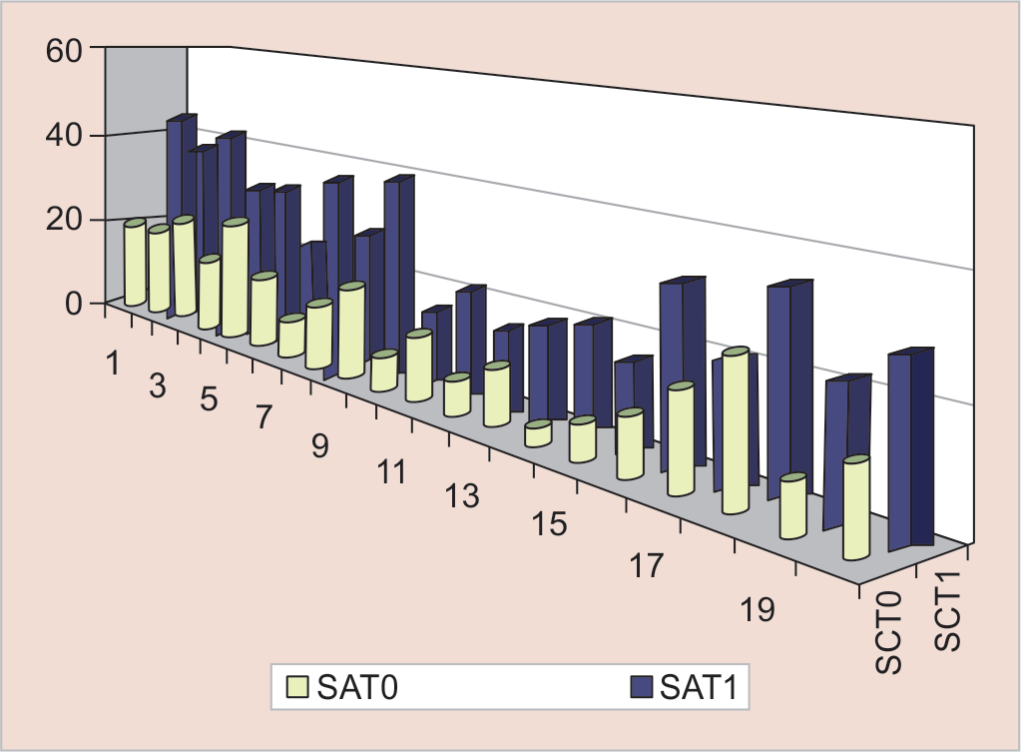
Comparison between SC pre- and postextraction

**Graph 3:**
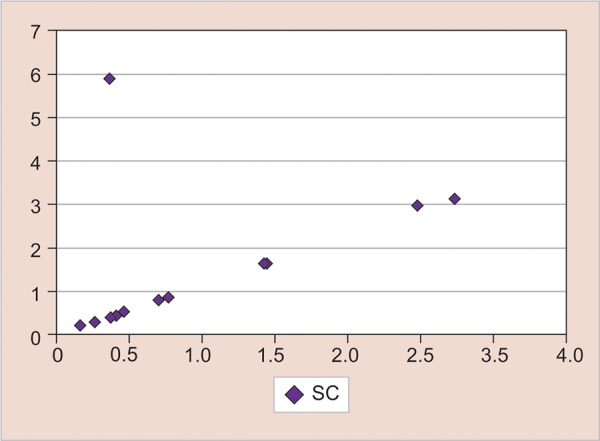
Pearson’s correlation between SAA and SC postextraction

Graph 2 shows the comparison between SCT0 (mean 15.4325, SD 0.876) and SCT1 (mean 27.18, SD 9.347367). The mean increase was 7.17, which was highly statistically significant (p = 0.00023 < 0.05). Graph 3 shows the correlation between SAT1 and SCT1 values postextraction (r = 0.1982).

The graph shows a straight line rising from left to right and it denotes a positive correlation between the two sets of data as the slope of the graph is positive.

## DISCUSSION

Dental environment is a new place for the child and therefore, it triggers stress among the children. Cortisol and amylase are important stress markers of the human body.

Upon comparison, a wide range of variability was noted in the cortisol and amylase levels of saliva in children pre- and postextraction. Salivary glands produce SAA,^7^ which helps in the digestion of starch.^8^ These glands have a large number of norepinephrine-stimulated beta-adrenergic receptors.^7,9^

Sympathetic adrenal medullary system (SAMS) activation that is a part of autonomic nervous system during psychological stress increases plasma norepinephrine, with a consequent increase in the production and release of SAA from the acinar cells of the parotid and subman-dibular salivary glands.^10,11^ Pain, a stressful agent,^12^ with SAMS activating capacity^13,14^ increases the production and secretion of alpha-amylase, and hence, can be used as a biomarker of pain sensation.^15,16^

Some recent studies show significant changes in SAA levels in children pre- and postdental treatment. This also indicates that anticipation of treatment may cause an increased SAA response.^17-19^ In response to stress, cortisol is also secreted, which enters into complex interactions with the hormonal and immune system.

It activates the hypothalamus-pituitary axis, which leads to production of the hormone ACTH which acts upon the adrenal cortex, leading to an increase in the levels of SC.^20^ As demonstrated by Miller et al,^21^ the level of SC was highest in patients undergoing tooth extraction when compared with other procedures, such as prophylaxis, restorative treatments, and examination. They evaluated the adrenal stress response to various dental treatments in healthy adults and found that cortisol levels measured at the start of a dental procedure decreased in patients undergoing noninvasive dental procedures, such as routine examinations. Conversely, cortisol levels at the end of tooth extractions were elevated compared with baseline cortisol.^17,20^

Padmanabhan et al^22^ in their study concluded that SC was higher in the study group and within the study group, it was raised when extractions were there than in the appointments in which oral prophylaxis and restorations were done respectively.

On the contrary, Kanegane et al^23^ found no correlation between SC concentrations and dental anxiety. Cortisol was raised only in patients who reported with pain. Similarly, Furlan et al^18^ did their study on the SC, alpha-amylase and heart rate variation in response to dental treatment in children and found higher cortisol and amylase levels before.

Sadi et al^20^ analyzed the levels of SC and SAA by salimetrics. A significant association was found between dental anxiety scores (DAS), pain, and past traumatic dental experience. However, a significant correlation was not established between DAS, SC, and SAA levels.

Our findings suggest that the SC and SAA levels are related to sympathetic nervous system activity. It was observed that there was an increase in the levels of both the markers postextraction in the subjects which can be justified as the anticipation of the treatment leads to precipitation of anxiety and stress.

Saliva used in the study serves as an easy, noninvasive method of analyzing the levels of stress markers when compared with serum which is an invasive method. A positive correlation between the stress, SAA, and SC was observed in our study, which is in contrast to the other studies and gives us an idea of further research in this field.

Although the study showed saliva as a new method of measuring anxiety and stress, it had no comparisons with the DAS. Further, it could also have been compared with the pain measuring scale. Another factor concerned was the high cost of the tests being performed for assessment of SC and alpha-amylase.

More studies of similar kind using different dental procedures on a larger population are needed to be conducted to better understand the correlation between these markers and stress. The study showed that the stress levels of a child increase during invasive procedures, such as tooth extraction. So, the use of some behavior modification and shaping techniques to overcome the anxiety of the child before commencement of the procedure is suggested to overcome the anxiety of the child before commencement of the procedure.

This can aid in better cooperation of the child during treatment as well as help in internal motivation toward future dental treatment.

## CONCLUSION

Assessment of SAA and SC can be an easy-to-use, non-invasive, and reliable biomarker for measuring stress in dental settings as:

1. Dental visit is associated with the presence of dental anxiety and stress in children.
2. Salivary cortisol and SAA are important stress markers of the human body.
3. Significant correlation is present between the stress, SC and SAA.

## CLINICAL SIGNIFICANCE

This study suggested that the child gets stressed during invasive procedures, such as tooth extraction, which implies that the dentists should use some behavior modification and shaping techniques to overcome the anxiety of the child before commencement of the procedure. This can aid in better cooperation during treatment as well as helps in internally motivating the child toward future dental treatment.
